# New Strategy for Preparation of Yttria Powders with Atypical Morphologies and Their Sintering Behavior

**DOI:** 10.3390/ma16072854

**Published:** 2023-04-03

**Authors:** Sheng Qu, Jinlian Li, Zhaoyang Liu

**Affiliations:** 1 School of Mechanical Engineering and Automation, Northeastern University, Shenyang 110819, China; 2 Iron and Steel Research Institute of Ansteel Group, Anshan 114051, China; 3 School of Metallurgy, Northeastern University, Shenyang 110819, China

**Keywords:** electric field, yttria, conventional sintering, spark plasma sintering, morphology

## Abstract

A modified precipitation method was used to prepare yttria powers for the fabrication of yttria ceramics in this study. The precipitation behavior, phase evolution, and shape of the yttria precursor were all examined in the presence or absence of an electric field. The findings demonstrate that the phases of the yttria precursor were Y_2_(CO_3_)_3_·2H_2_O with and without an electric field, while the morphology changed from flake to needle-like under the action of the electric field. After calcining both yttria precursors at 750 °C, yttria powders with similar morphologies were obtained and then densified via conventional sintering (CS) and spark plasma sintering (SPS). The densification and thermal shock resistance of the yttria ceramics were investigated. The yttria ceramics sintered using SPS had higher bulk density and thermal shock resistance than the samples sintered using CS. When the sintering process for the ceramics sintered from needle-like yttria powder was switched from CS to SPS, the bulk density increased from 4.44 g·cm^−3^ to 5.01 g·cm^−3^, while the number of thermal shock tests increased from two to six. The denser samples showed better thermal shock resistance, which may be related to the fracture mechanism shifting from intergranular fracture to transgranular fracture.

## 1. Introduction

Due to its high-temperature strength and high corrosion resistance, yttria (Y_2_O_3_) is widely used in the preparation of various high-grade materials [1,2,3], such as transparent ceramics for laser crystals and crucibles for titanium alloy melting [4,5].To achieve excellent properties, high-density yttria materials are typically prepared through sintering or electric melting [6,7]. In the sintering process, the main factors affecting the densification of yttria are the raw material properties as well as the sintering process and methods [8,9]. At present, there are many methods to prepare yttria powders, such as precipitation, decomposition, combustion, and solvothermal techniques [10,11,12,13,14]. Although high-purity yttria powders can be prepared using these methods, no new technologies have been developed within these methods to further improve and control the performance of the resulting powders. Moreover, the majority of Y_2_O_3_ powders that researchers have investigated are spherical. Spheres are the most typical and ideal shape for densification sintering. However, there are few studies on the preparation and sintering of one- and two-dimensional Y_2_O_3_ powders, such as powders with flake and needle-like morphologies. Changing the powder morphology will affect the sintering behavior as well as the physical and chemical properties of sintered ceramics. Special consideration should be given to the various preparation process variables, such as reaction temperature, raw material composition and molar ratio, and reaction and aging time, to produce these anisotropy powders.

In recent years, electric current pulse technology has been widely used for material preparation, especially to control the microstructure and refine the particle size of metal materials during solidification [15,16,17]. Crystallinity and particle size are important factors affecting the properties of powders, especially their sintering properties [18,19]. Therefore, applying a pulsed electric current during the preparation of yttria powders is expected to influence their crystallinity and particle size. Many methods have been adopted for the sintering of yttria, such as conventional sintering (CS) [20], hot pressing sintering [21], microwave sintering [22], and spark plasma sintering (SPS) [23]. However, it is difficult to densify yttria, so obtaining good densified yttria materials using conventional processes is a significant challenge [24,25,26]. Therefore, new yttria sintering technologies should be adopted to promote its sintering and densification. The SPS approach is considered the most innovative sintering method because it allows for a wide range of sintering parameters (such as temperature, pressure, and holding time) to be varied within an electro-physical treatment. This allows the morphology of composite ceramics to be tuned in a wide range [27]. Spark discharge occurs between particles when a pulsed electric current is applied to graphite molds and powders. As a result, the residual gas in the pores is broken down, and plasma is formed, promoting local bonding between grains. Furthermore, the high-pressure and vacuum conditions during the sintering process promote gas discharge, reduce the gap between powders, and promote powder rearrangement. These favorable conditions ensure that SPS can achieve powder densification and sintering in a short period of time.

Based on the sintering characteristics of yttria and its importance in the field of materials science, in this study, yttria powders were prepared by precipitation method, with and without the application of a pulsed electric current. The sintering process of yttria, the influence of this process on the crystallinity and morphology of the obtained yttria powders, and the sintering behavior were investigated.

## 2. Materials and Methods

### 2.1. Preparation of Yttria Powders

**Figure 1:**
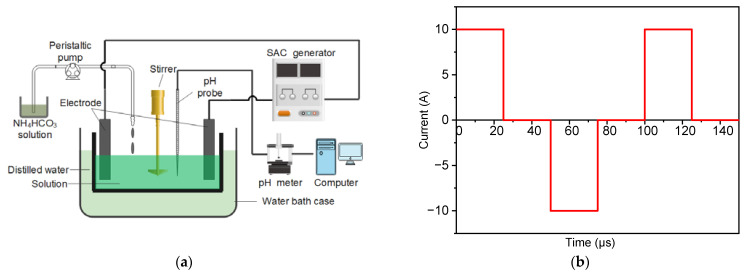
(**a**) Preparation process of yttria powders and (**b**) waveform of the applied current.

Yttrium nitrate hexahydrate (Y(NO_3_)_3_·6H_2_O, 5N purity) and ammonium hydrogen carbonate (NH_4_HCO_3_, AR grade) were used as raw materials to prepare yttria powders. The process flow is shown in Figure 1a. Y(NO_3_)_3_·6H_2_O and NH_4_HCO_3_ were separately dissolved in deionized water to form two solutions with concentrations of 0.1 mol·L^−1^ and 1.5 mol·L^−1^, respectively. Subsequently, the NH_4_HCO_3_ solution was added dropwise to the Y(NO_3_)_3_·6H_2_O solution using a peristaltic pump at a flow of 5 mL·min^−1^. A mechanical stirrer (500 rpm) was used in the precipitation process to evenly mix the solution. A pH probe (pHS-3C) was calibrated and fixed in the beaker to measure the pH of the solution in situ throughout the precipitation process. Subsequently, the yttria precursor was washed three times with deionized water and then dried at 70 °C for 48 h. After drying, the yttria precursor powders were calcined at different temperatures for 60 min to obtain the yttria powders. For comparison, yttria precursor powders were also prepared under similar conditions using electric current pulses. Symmetric positive and negative electric pulses with a frequency of 10 kHz were applied during the precipitation process, and the waveform of the applied current is shown in Figure 1b.

### 2.2. Sintering of Yttria

**Figure 2:**
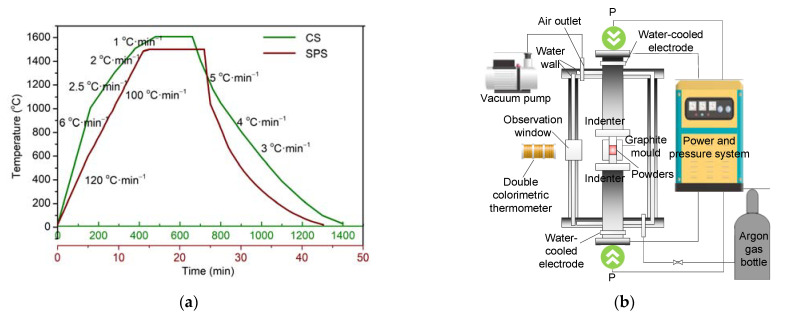
(**a**) CS and SPS temperature program (**b**) schematic diagram of SPS equipment.

The obtained yttria powders were sintered using two sintering methods: CS and SPS. In the CS process, yttria powders were first pressed into cylindrical samples (Φ10 × 6.5 mm^2^) under a pressure of 200 MPa. Then, these samples were placed into a muffle furnace and heated from room temperature to 1600 °C for 3 h (soaking time). The detailed heating and cooling rates are shown in Figure 2a. In the SPS process (LABOXTM-210), the sintering equipment was composed of the furnace body, power and pressure system, temperature measurement system, and water-cooling system, as shown in Figure 2b. A graphite die was used for forming and heating, and the pressure in the furnace was less than 5 Pa. The yttria powders were added to the graphite die and heated to 1500 °C at 100–120 °C/min under a pressure of 50 MPa. The powders were held under these conditions for 10 min.

### 2.3. Characterization

The bulk density and apparent porosity of the yttria ceramics were determined via the Archimedes principle using deionized water. Linear shrinkage was determined by measuring the diameter and height of each sample before and after firing using a caliper with a precision of ±0.02 mm. The thermal shock resistance of the samples was tested using the following method: each sample was placed in an electric furnace at 800 °C and held at this temperature for 10 min. Subsequently, the sample was taken out of the electric furnace, cooled in water for 3 min, and then cooled down in air for 12 min. This heating and cooling process was repeated several times until the sample cracked. The number of cycles required for the sample to start to crack was taken as an index for evaluating the thermal shock resistance.

Thermogravimetry (TG) and differential scanning calorimetry (DSC) analyses were performed using a thermal analyzer (SETSYS18, Setaram, Caluire, France) under an air atmosphere at a heating rate of 5 °C·min^−1^ over the temperature range of 50–1000 °C. The phase composition of the yttria precursor as a function of firing temperature was determined via X-ray diffraction (XRD, Philips PW3040/60, Amsterdam, The Netherlands) measurements using a Cu Kα (λ = 0.154056 nm) radiation source operated at 40 kV and 40 mA. The angular scan range was 2θ = 10–70°, and the scan rate was 5°/min. After the thermal shock tests, the microstructure of the samples was observed via scanning electron microscopy (SEM).

## 3. Results and Discussion

**Figure 3:**
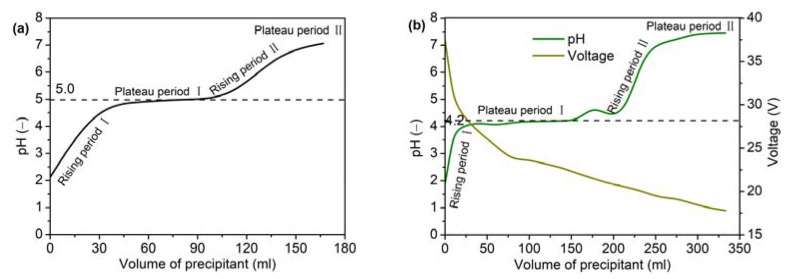
Change in pH and voltage with precipitant volume: (**a**) without electric field and (**b**) with electric field.

During the preparation of the yttria precursor via the precipitation method, the pH of the mixed solution changed as the NH_4_HCO_3_ solution was added to the Y(NO_3_)_3_ solution. It should be noted that the pH of the Y(NO_3_)_3_ solution was approximately 2, not neutral. This was mainly due to the presence of residual acid in the production of Y(NO_3_)_3_·6H_2_O. As shown in Figure 3a, the evolution of the mixed solution pH was divided into four stages when no electric field was applied: two stages in which the pH increased and two stages in which the pH plateaued. First, the pH value of the mixed solution gradually increased as the NH_4_HCO_3_ solution was added dropwise (Rising stage I). In this stage, NH_4_HCO_3_ mainly neutralized the residual acid in the yttrium nitrate solution, and almost no precipitation occurred. With the further addition of NH_4_HCO_3_ solution, the mixed solution entered the first plateau region (Plateau stage I), and the pH value in this stage was around 5.0. At this time, a large amount of white precipitate was generated in the mixed solution, and the NH_4_HCO_3_ mainly reacted with the yttrium ions in the solution. When no yttrium ions remained in the solution, NH_4_HCO_3_ was in excess, and the pH of the mixed solution entered the second rising stage (Rising stage II). Finally, the pH of the mixed solution approached the pH of the NH_4_HCO_3_ solution and stopped rising (Plateau region II). When the electric field was applied, the pH evolution was similar to that achieved without the electric field, but the corresponding pH value of the first plateau region was lower (around 4.2). Xu et al. [28] prepared a yttria precursor through a precipitation method using ammonia as the precipitant, and they also found that the pH of the mixed solution decreased after the application of an electric field. This indicated that under the action of an electric field, NH_4_HCO_3_ could react with yttrium ions at a lower pH value and promote the formation of the precipitate. Additionally, the total ion concentration in the mixed solution increased with the addition of the NH_4_HCO_3_ solution, and the voltage decreased when a steady electric current was applied, as shown in Figure 3b.

**Figure 4:**
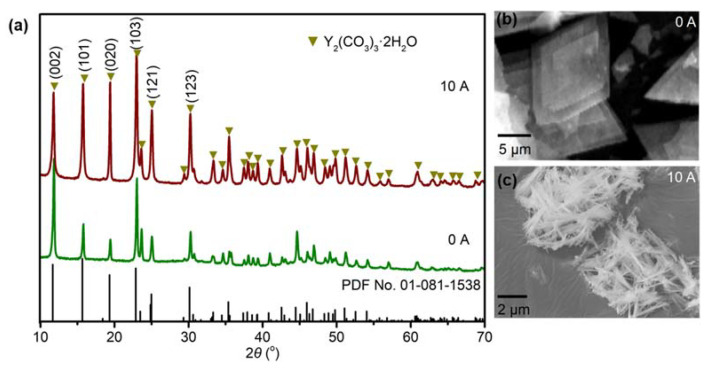
(**a**) XRD patterns and (**b**,**c**) SEM images of yttria precursor with and without the application of an electric field.

Yttrium carbonate can exist in two forms: Y_2_(CO_3_)_3_ and Y(OH)CO_3_. The type of precipitate generated depends on the type and concentration of ions present in the precipitant. Zhang et al. [29] indicated that the obtained precipitate changed from Y(OH)CO_3_ to Y_2_(CO_3_)_3_ when the molar ratio of Y(NO_3_)_3_·6H_2_O to urea was changed from 1:1 to 1:3. Based on HSAB (hard/soft acid/base) theory [30], CO_3_^2−^ and OH^−^ are hard bases and Y^3+^ is a hard acid. The polarity, ion charge, and ionic radius values of these species mean that they easily react to form Y(OH)CO_3_. However, when a large excess of NH_4_HCO_3_ is present, a high level of CO_3_^2−^ is obtained in the system. As a result, Y_2_(CO_3_)_3_·2H_2_O is formed instead of Y(OH)CO_3_. As shown in the XRD patterns in Figure 4a, Y_2_(CO_3_)_3_·2H_2_O was generated both with and without an electric field, which was mainly due to the high molar ratio of NH_4_HCO_3_ to yttrium nitrate in the preparation process. Equations (1)–(4) show the hydrolysis equilibrium of NH_4_HCO_3_ in water, and the total reaction for the formation of Y_2_(CO_3_)_3_·2H_2_O is shown in Equation (5). Although Y_2_(CO_3_)_3_·2H_2_O was generated in both the absence and presence of an electric field, the intensity of its diffraction peaks changed. When NH_4_HCO_3_ was added to the solution without an electric field, the peaks corresponding to the (002) and (103) crystal planes were more intense. However, when an external electric field was applied, the intensities of the peaks corresponding to the (101) and (020) crystal planes were higher. The changes in the relative intensities of these diffraction peaks may be mainly related to the change in morphology of the yttria precursor. As shown in Figure 4b,c, needle-like and flake yttria precursor morphologies were generated by performing the precipitation reaction with and without an electric field, respectively. Meanwhile, the peak intensities of Y_2_(CO_3_)_3_·2H_2_O prepared under an electric field were higher than those of the sample prepared without an electric field. This may indicate that higher powder crystallinity was achieved under the action of the symmetric positive and negative electric pulses. The main reason for this is likely the occurrence of Y^3+^ and CO_3_^2−^ ion micro-vibrations during the formation of Y_2_(CO_3_)_3_·2H_2_O under the action of the electric field.

NH_4_HCO_3_ + H_2_O = NH_4_OH + H_2_CO_3_, (1)

NH_4_OH = NH_4_ + OH^−^, (2)

H_2_CO_3_ = H^+^ + HCO_3_^−^, (3)

HCO_3_ = H^+^ + CO_3_^2−^, (4)

2Y(NO_3_)_3_ + 6NH_4_HCO_3_ + 2H_2_O = Y_2_(CO_3_)_3_·2H_2_O + 6NH_4_NO_3_ + 3CO_2_↑ + 3H_2_O, (5)

**Figure 5:**
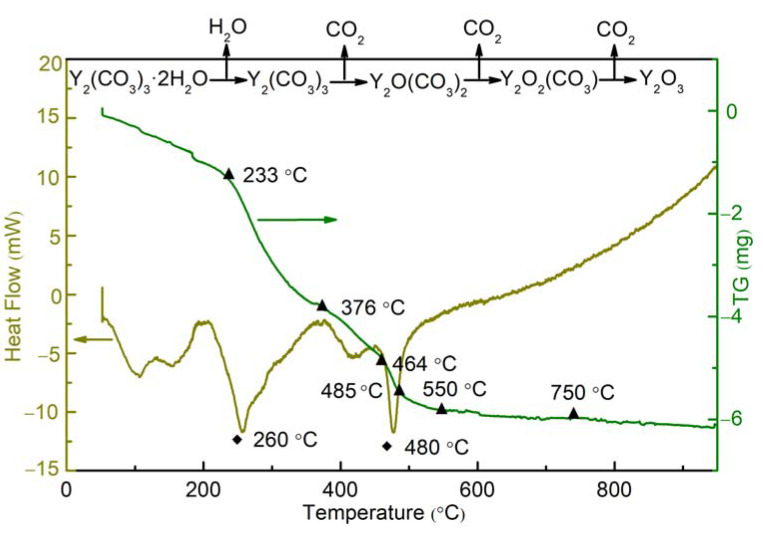
TG-DSC curves of yttria precursor.

To clarify the decomposition process of Y_2_(CO_3_)_3_·2H_2_O and determine the final calcination temperature, the samples were thermally characterized by TG and DSC analysis. The process occurring during heating was predicted, and the critical temperature of new phase formation was determined. Figure 5 shows the TG–DSC curves for Y_2_(CO_3_)_3_·2H_2_O. Crystal water was vaporized, and Y_2_(CO_3_)_3_ was formed in the temperature range of 55 −233 °C. At 233–376 °C, Y_2_(CO_3_)_3_ decomposed into Y_2_O(CO_3_)_2_, and a very intense endothermic peak was observed at around 260 °C. The weight loss between 367 °C and 464 °C indicated that Y_2_O(CO_3_)_2_ decomposed to form Y_2_O_2_(CO_3_). Above 464 °C, Y_2_O_2_(CO_3_) continued to decompose into Y_2_O_3_, and an intense endothermic peak appeared at around 480 °C. As the temperature increased to 750 °C, the TG curve remained basically unchanged, indicating that Y_2_(CO_3_)_3_·2H_2_O was completely decomposed into Y_2_O_3_.

**Figure 6:**
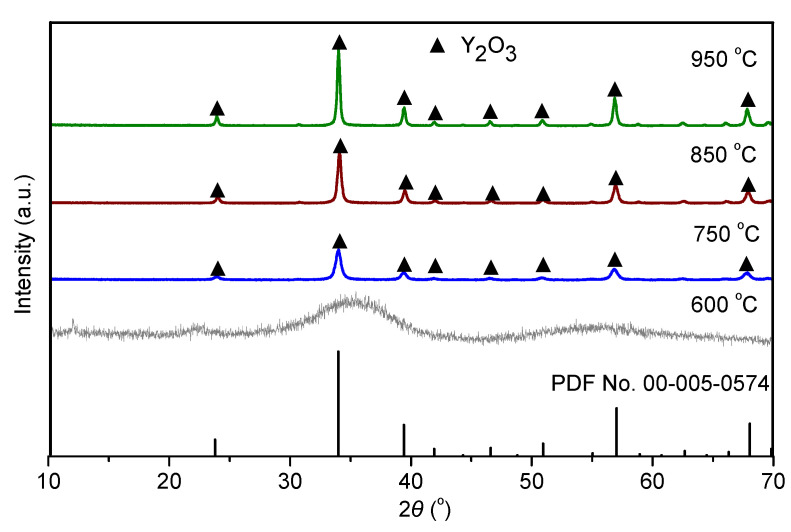
XRD patterns of yttria precursor after calcining at different temperatures.

Figure 6 shows the XRD patterns depicting the evolution of the mineralogical phases of the Y_2_(CO_3_)_3_·2H_2_O samples after firing at different temperatures. After calcination at 600 °C, no clear diffraction peak was observed in the XRD pattern, indicating that the product was amorphous. Peaks corresponding to the Y_2_O_3_ phase were observed in the XRD patterns of the samples calcined in the temperature range of 750–950 °C, and more intense peaks were observed at higher temperatures. This is because, at low calcination temperatures, yttria particles were in the early stage of structure formation. As the calcination temperature increased, more crystal growth occurred [31]. Due to the low energy and poor particle diffusion capacity, it was difficult for crystal nuclei to grow, leading to small grain size. The TG–DSC curves show that the optimal calcination temperature of the Y_2_(CO_3_)_3_·2H_2_O precursor was 750 °C.

**Figure 7:**
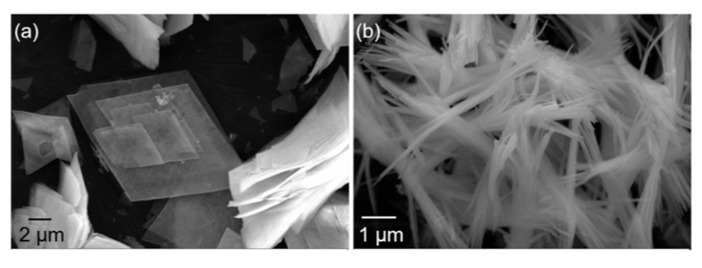
SEM images of yttria with different morphologies: (**a**) flake morphology obtained in the absence of an electric field; (**b**) needle-like morphology obtained in the presence of an electric field.

Figure 7 shows the morphology of Y_2_O_3_ powders after firing at 750 °C with and without the application of an electric field during the precipitation process. Compared to the precursors, the morphologies of the calcined Y_2_O_3_ powders did not significantly change, with only a slight increase in dimensions. The yttria powders prepared without an electric field were mainly composed of flakes with a side length of a few tens of microns and a thickness of a few hundred nanometers. Zhang et al. [32] found that the connection orientation between grains does not change during the decomposition of precursors. Therefore, the shape and size of precursors remain largely unchanged before and after calcination. Under the action of the electric field, the morphology of the Y_2_O_3_ powders changed to needle-like, with needles of about a dozen microns in length and a few hundred nanometers in diameter. This shift in morphology may be related to the lower pH corresponding to yttria precursor nucleation (Figure 3).

**Figure 8:**
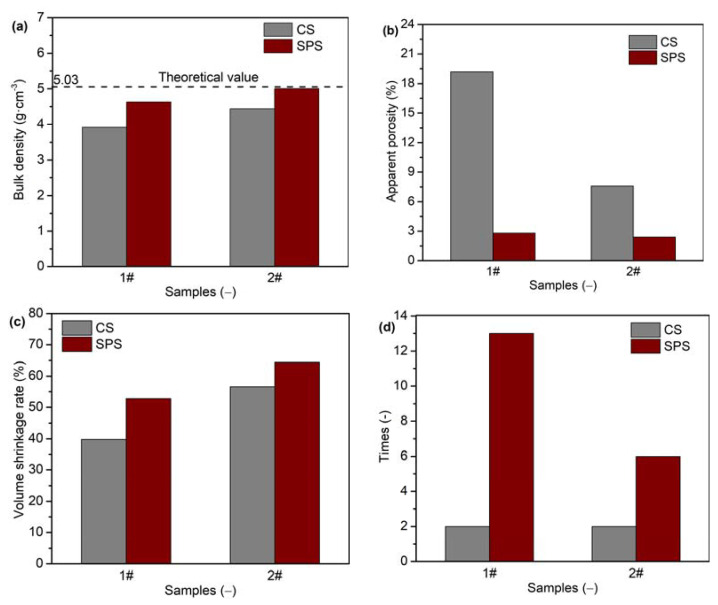
(**a**) Bulk densities, (**b**) apparent porosities, (**c**) volume shrinkage rates, and (**d**) number of thermal shock cycles of yttria ceramics.

The yttria powders with flake (sample 1#) and needle-like (sample 2#) morphologies were sintered via CS and SPS. The bulk density, apparent porosity, volume shrinkage rates, and number of thermal shock cycles of the obtained yttria ceramics are shown in Figure 8. It can be clearly seen that, although the CS process used a higher pressure (100 MPa) and sintering temperature (1600 °C) as well as a longer holding time (180 min), the bulk density and volume shrinkage rate of the yttria ceramics sintered via CS were lower than those of the samples sintered via SPS. Furthermore, the apparent porosity of the ceramics sintered via CS was accordingly higher. It was difficult to sinter the dense yttria powder (sample 1#) with a flake morphology using CS. The apparent porosity was as high as 19.17%, and the bulk density was only 3.92 g·cm^−3^. However, when using SPS, the density considerably improved to 4.63 g·cm^−3^, and the apparent porosity was reduced to 2.83%. For sample 2#, the apparent porosity of yttria ceramic sintered via CS was as high as 7.58%, while that of SPS-sintered ceramic was only 2.42%. The bulk density and relative density of sample 2# obtained using CS were only 4.44 g·cm^−3^ and 88.26%, respectively. Meanwhile, bulk density and relative density values of 5.01 g·cm^−3^ and 99.60% were, respectively, achieved when using SPS. This bulk density value is very close to the theoretical value (5.03 g·cm^−3^). In the SPS process, a pulsed current is directly applied to the graphite mold and yttria powder. When the gap between grains is discharged, the local temperature may increase by up to several thousand degrees or even tens of thousands of degrees. This causes evaporation and melting on the grain surface, and a neck is formed at the contact point between grains, which promotes sintering. In addition, yttria powders are always held under pressure during the sintering process, which promotes the discharge of gas, reduces the gap between powders and promotes their rearrangement [33,34]. Moreover, the chamber is constantly under vacuum, which also promotes the expulsion of gas between particles. These favorable conditions accelerate the densification process.

The bulk density of sample 1# was higher than that of sample 2# for both the CS and SPS processes, indicating that the yttria powders with needle-like morphology were easier to densify than the yttria powders with flake morphology. The thermal shock test results showed that yttria ceramics had better thermal shock properties when prepared via SPS rather than CS. When sintered via SPS, samples 1# and 2# were able to undergo thirteen and six thermal shock cycles, respectively; these numbers were much larger than those of the corresponding samples sintered via CS. It is interesting to note that the denser samples exhibited better thermal shock properties.

As diffusion progresses, atoms and vacancies are transported along the surface, interface, or body of materials during solid-phase sintering. The difference in free energy or chemical potential between the free surface of particles and the contact interface with adjacent particles is the most important driving force for solid-phase sintering. Kingery derived the following expression for solid-phase sintering [35]:

Δ*L*/*L* = (20*γa*^3^D*/(k*T*) × 2^1/2^)^2/5^ × *r*^−6/5^ × *t*^2/5^, (6)

where Δ*L/L* is the linear shrinkage, which is equivalent to the sintering rate, %; *a*^3^ is the atomic volume of the diffusion vacancy, m^3^; *k* is the Boltzmann constant, 1.38 × 10^−23^ J·K^−1^; t is the time of sintering, s; *γ* is the surface energy, J·m^−2^; D* is the diffusion coefficient, m^2^; *T* is the temperature, *K*; and *r* is the particle diameter, m.

Equation (6) shows that the sintering rate gradually decreases with time and has a midpoint density. Therefore, it is difficult to improve the properties of a sintered sample by only extending the time. The sintering rate is roughly inversely proportional to the particle size—that is, the smaller the sintered particle size, the higher the sintering rate.

**Figure 9:**
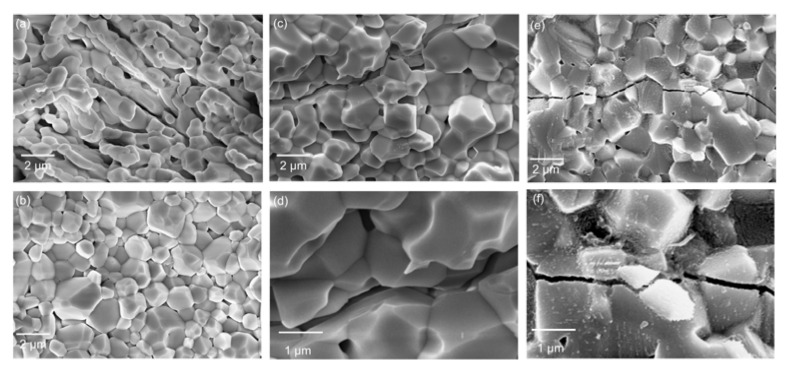
SEM images of yttria ceramics after thermal shock test: (**a**) sample 1#, CS; (**b**) sample 1#, SPS; (**c**,**d**) sample 2#, CS; (**e**,**f**) sample 2#, SPS.

The thermal and mechanical properties of ceramics can be comprehensively reflected by their thermal shock resistance [36]. Based on the unified theory of brittle ceramics proposed by Hasselman [37], the thermal shock resistance of ceramics can be improved by reducing the coefficient of thermal expansion and improving the resistance to crack initiation and propagation [38]. Figure 9 shows SEM images of the cross-section of yttria samples after thermal shock resistance tests. The sample sintered via CS possessed more pores than the sample sintered via SPS. No evident cracks were observed in these images for either sample. Similar to the results obtained for sample 1#, the porosity of sample 2# sintered via SPS was significantly lower than that of the sample sintered via CS. The crack caused by thermal shock visible in the cross-section of the sample sintered via CS indicated intergranular fracture. In contrast, the fracture mode was transgranular for the SPS-sintered sample. Zhang et al. [39] indicated that a large number of sintering necks strengthens the connection between grains, meaning that the ceramics can exhibit obvious transgranular fracture under external force. This fracture mechanism is favorable evidence for enhancing room-temperature flexural strength. In general, more energy is required for a transgranular fracture to occur, explaining the higher thermal shock resistance of the SPS-sintered sample.

## 4. Conclusions

Yttria powders with flake and needle-like morphologies were synthesized by precipitation method, with and without the application of an external electric field. Yttria ceramics were then fabricated using the two powders as raw materials via CS and SPS. The preparation process and properties of yttria powders and ceramics were investigated, and the main conclusions are as follows:

1. The application of an electric field during the precipitation process promoted the nucleation of yttria precursors at a lower pH, resulting in a morphological transformation from flake to needle-like.
2. Compared to yttria ceramics prepared via CS, yttria ceramics prepared via SPS had higher density and lower porosity. This was true for both the flake and needle-like morphologies. The relative density of the yttria ceramic sample 2# reached 99.60% after sintering via SPS.
3. The number of thermal shock cycles increased from two to six in sample 2# when the sintering method changed from CS to SPS. This is mainly attributed to the transformation of the fracture mode from intergranular to transgranular.

## Data Availability

Not applicable.
